# Non-genomic effects of nuclear receptors: insights from the anucleate platelet

**DOI:** 10.1093/cvr/cvy044

**Published:** 2018-02-14

**Authors:** Amanda J Unsworth, Gagan D Flora, Jonathan M Gibbins

**Affiliations:** School of Biological Sciences, Institute of Cardiovascular and Metabolic Research, Harborne Building, Whiteknights, Reading RG6 6AS, Berkshire, UK

**Keywords:** Nuclear receptors, Non-genomic, Platelets, Thrombosis

## Abstract

Nuclear receptors (NRs) have the ability to elicit two different kinds of responses, *genomic* and *non-genomic*. Although genomic responses control gene expression by influencing the rate of transcription, non-genomic effects occur rapidly and independently of transcriptional regulation. Due to their anucleate nature and mechanistically well-characterized and rapid responses, platelets provide a model system for the study of any non-genomic effects of the NRs. Several NRs have been found to be present in human platelets, and multiple NR agonists have been shown to elicit anti-platelet effects by a variety of mechanisms. The non-genomic functions of NRs vary, including the regulation of kinase and phosphatase activity, ion channel function, intracellular calcium levels, and production of second messengers. Recently, the characterization of mechanisms and identification of novel binding partners of NRs have further strengthened the prospects of developing their ligands into potential therapeutics that offer cardio-protective properties in addition to their other defined genomic effects.

## 1. Introduction

Nuclear receptors (NRs) represent the family of mammalian proteins associated with the transcriptional regulation in human tissues and include the androgen receptor (AR), oestrogen receptor (ER), glucocorticoid receptor (GR), farnesoid X receptor (FXR), liver X receptor (LXR), peroxisome proliferator-activated receptors (PPARs), retinoic acid receptor (RAR), retinoid X receptor (RXR), and the vitamin D receptor (VDR). Upon activation by their lipophilic ligands, NRs regulate several fundamental biological processes, such as cell proliferation, differentiation, metabolism, and homeostasis (*Table 1*).^1^^,^^2^ Any deviation from their normal function can lead to the pathological manifestations, such as cancer, diabetes, arthritis, and obesity.^3^

**Table 1:** NRs and their biological functions

Nuclear receptor	Ligands	Biological function
GR	Natural: glucocorticoid	Lipolysis Glucose metabolism
	Synthetic: RU38486, A348441	

ER	Natural: oestrogen, including oestrone (E1), oestradiol (E2) and oestriol (E3)	Development of the female reproductive system and secondary sexual characteristics

AR	Natural: dihydrotestosterone, testosterone	Development of the male reproductive system and secondary sexual characteristics
	Synthetic: mibolerone	

LXR	Natural: oxysterols	Lipid and carbohydrate metabolism
	Synthetic: T0901317, GW3965	

FXR	Natural: bile acids	Bile acid homeostasis
	Synthetic: GW4064, farnesol, CDCA	

PPARα	Natural: polyunsaturated fatty acids	Fatty acid oxidation and lipid metabolism
	Synthetic: fibrates (gemfibrozil, fenofibrate, clofibrate)	

PPARβ	Natural: unsaturated/saturated fatty acids, eicosanoids, prostacyclin	Cholesterol metabolism
	Synthetic: GW501516	

PPARγ	Natural: 15-deoxy-12, 14 prostaglandin J2 (15d-PGJ2)	Lipid and glucose metabolism
	Synthetic: thiazolidinedione (ciglitazone, pioglitazone, rosiglitazone)	

RAR	Natural: atRA	Cell growth, differentiation and organogenesis

RXR	Natural: 9-*cis*-retinoic acid, docosahexaenoic acid	Cellular proliferation and differentiation, glucose, fatty acid and cholesterol metabolism
	Synthetic: methoprene acid, rexinoids (LG100268)	

VDR	Natural: calcitriol	Calcium homeostasis, cell proliferation and differentiation
	Synthetic: maxacalcitol, calcipotriol	

NRs have the ability to function in both *genomic* and *non-genomic* ways. Whilst historically associated with the regulation of transcription and control of gene expression (genomic), more recently non-genomic roles for the NRs have been identified that occur independently of transcriptional regulation. Unlike the genomic functions, which can occur over minutes or hours, these events occur in the time frame of seconds to a few minutes, which is considered too rapid to be attributed to the biosynthesis of mRNA or protein, and is often unaffected by the inhibitors of transcription or translation.^4^ The non-genomic functions of NRs vary^5–7^ and whilst it is thought these functions are initiated by physical interactions of NRs with cofactors and binding partners that initiate rapid signalling events, the exact mechanisms are not well understood. One possible explanation is that the cellular localization of NRs influences the availability of cofactors and substrates, which leads to varying combinations of binding partner interactions. For instance, localization of NRs to the cytosol, plasma membrane, or other intracellular organelles such as mitochondria increases the likelihood of initiation of non-genomic effects, whilst genomic functions may be restricted to when NRs are localized in the nucleus.^8–11^ The formation of different multi-protein signalling complexes with different localization and distribution patterns across different cell types could offer a high degree of cell and tissue selective action for the NRs but as of yet these are poorly defined.^10^^,^^11^ Given the underlying differences between the genomic and non-genomic activities, non-genomic effects are more easily observed in cell types that lack a functional nucleus such as erythrocytes and platelets.

Emerging evidence indicates that platelets are also involved in roles beyond those described in haemostasis and thrombosis. For instance, granule secretion following platelet activation results in the release of an array of chemokines, cytokines, growth factors, anti-inflammatory factors, and several other biologically active molecules into the vicinity of injured tissues that contribute towards the progression of numerous diseases including inflammatory conditions (e.g. atherosclerosis and rheumatoid arthritis),^12^^,^^13^ type 2 diabetes,^14^ and cancer cell metastasis).^15^ Thus, platelets are highly active cells with diverse functions despite lacking genomic DNA. Although devoid of a nucleus, platelets still contain different forms of RNA (mRNA, rRNA, tRNA, and miRNA) and components of the transcription and translation machinery that are derived from megakaryocytes during thrombopoiesis.^16^ There is a growing consensus that these RNAs are not subjected to a random transfer by megakaryocytes but are specifically sorted and are competent for translation within platelets.^17^^,^^18^ Moreover, there is evidence to suggest that platelet-derived microparticles may deliver platelet mRNAs into other nucleated cells, such as monocytes and endothelial cells, where they then undergo translation.^19^ Components of this transcription machinery found to be present inside platelets include the intracellular NRs. Due to their anucleate nature and mechanistically well characterized and rapid responses, such as aggregation and adhesion, platelets provide an excellent model system to study the acute non-genomic effects of the NRs.^20^^,^^21^

## 2. NRs are acute regulators of platelet function

On the basis of the mechanisms of action in the nucleated cells, NRs are classified into two classes: type I or the steroid hormone receptors and type II or the non-steroid receptors. Platelets are known to express both the classes of these receptors. This includes the AR,^22^^,^^23^ ER,^22^^,^^24–26^ GR,^27^^,^^28^ FXR,^29^^,^^30^ LXR,^30^^,^^31^ PPARs,^32–40^ RAR,^41^ RXR,^42^^,^^43^ and VDR.^44^^,^^45^ Both natural and synthetic ligands for these NRs have been shown to alter platelet function through a variety of mechanisms as described below and summarized in *Table 2*.

**Table 2:** A summary of NRs identified in platelets and their modes of action

Nuclear receptor	Ligands	Effect on platelet function	Mechanisms of action
GR^27^^,^^28^	Prednisolone	Negative regulation of platelet secondary mediator regulated effects (ADP and TXA_2_) In vitro Human platelets	Mechanism is unknown

ER^25^^,^^26^	Oestrogen—oestrone (E1), oestradiol (E2) and oestriol (E3)	Reduction in platelet responsiveness however, conflicting results exist In vitro, ex vivo, in vivo Human, mouse platelets	Mechanism is unknown

AR^46–48^	Testosterone Dihydrotestosterone	Potentiation of platelet aggregation *In vitro* and *ex vivo* Human and rat platelets	Mechanism is unknown

LXR^31^	GW3965 T0901317 24(S)-OH-cholesterol 27-OH-cholesterol	Inhibition of platelet function and thrombosis *In vitro* and *in vivo* Human and mouse platelets Conversion of platelets to the procoagulant state In vitro Human platelets	Reduced phosphorylation of early GPVI signalling components—Syk, LAT and PLCγ2 Increase LXR-Syk and LXR-PLCγ2 interaction Formation of coated platelets, including PS exposure, mitochondrial membrane depolarization (see *Figure 1*)

FXR^29^	GW4064 Chenodeoxycholic acid 6α-ethyl-chenodeoxycholic acid	Inhibition of platelet function, thrombosis and haemostasis *In vitro* and *in vivo* Human, mouse platelets	Cyclophillin D-dependent formation of coated platelets and closure of surface integrins Associated with PS exposure and mitochondrial membrane depolarization Augmented cGMP levels which promote PKG activity and phosphorylation of VASP S239 (see *Figure 1*)
		Conversion of platelets to the procoagulant state In vitro Human platelets	

PPARα^33^	Fenofibrate Statins	Inhibition of platelet function In vitro Human, mouse platelets	Increase in cAMP levels PPARα–PKCα interaction and attenuation of PKCα (see *Figure 2*)

PPARβ/δ^35^	GW0742 L-165041	Inhibition of platelet function In vitro Human, mouse platelets	Increase in cAMP levels PPARα–PKCα interaction and attenuation of PKCα (see *Figure 2*)

PPARγ^38^^,^^39^	15d-PGJ2 Thiazolidinediones (rosiglitazone, ciglitazone, pioglitazone)	Inhibition of platelet function, thrombosis and haemostasis *In vitro* and *in vivo* Human, mouse platelets	Inhibition in phosphorylation of Syk and LAT to reduce GPVI signalling Reduced PPARγ–Syk and PPARγ–LAT interaction upon PPARγ ligand treatment Negative regulation of integrin αIIbβ3 outside-in via up-regulation of PKA activity and inhibition β3 phosphorylation (see *Figure 2*)

RAR^41^	atRA	Inhibition of cytoskeletal rearrangements and platelet spreading In vitro Human platelets	Disruption of RARα–Arp2/3 interactions. (see *Figure 3*)
RXR^42^^,^^43^	9-*cis*-retenoic acid Methoprene acid Docosahexaenoic acid	Inhibition of platelet function, thrombosis and haemostasis *In vitro* and *in vivo* Human, mouse platelets	RXR–Gq interaction and negative regulation of Rac activation to inhibit GPCR-mediated platelet activation Up-regulation of PKA activity and phosphorylation of VASP S157 in cAMP- and NFκβ-dependent manner (see *Figure 3*)
VDR^45^	Vitamin D and its metabolites	Low vitamin D plasma levels cause high mean platelet volume, a marker of platelet hyperactivity In vivo Human platelets	Mechanism is unknown

## 3. Type I NRs

Mechanisms by which type I NRs (GR, ER, and AR) regulate platelet functions are poorly understood. This might be attributed to the variations in the plasma levels of the steroid hormones targeting these NRs (especially in females and under certain pathological conditions).^49–51^ This might lead to an inaccurate assessment of the role type I NRs play in modulating platelet functions in acute vs. chronic studies and might account for the existing contradictory published data. The effects of ligands of type 1 NRs on platelet function currently published are described below.

### 3.1 Glucocorticoid receptor

GRs are activated by glucocorticoid and anti-inflammatory hormones that regulate inflammation and glucose homeostasis.^52^ Prednisolone, a synthetic glucocorticoid derived from cortisol, has been shown to attenuate platelet function.^27^^,^^28^ Prednisolone-treated platelets displayed reduced aggregation and thromboxane B_2_ (TxB_2_) release in response to stimulation by either ADP or the TxA_2_ mimetic U46619, which was reversed following treatment with a GR antagonist mifepristone.^27^ This inhibition was not found to be associated with up-regulation of cyclic nucleotides—cyclic adenosine monophosphate (cAMP) or cyclic guanosine monophosphate (cGMP), key inhibitory mediators of platelet activity.^28^ Platelet adhesion and thrombus formation under flow on collagen *in vitro* were also found to be diminished following prednisolone treatment which is likely an outcome of reduced platelet responses to ADP and TxA_2_, secondary mediators of platelet activation that support platelet adhesion and thrombus growth.^28^ Prednisolone has also shown to modulate platelet–monocyte interactions following stimulation by ADP, which is attributed to an attenuation of platelet activity and not to inhibition of monocytes.^28^ It should be noted, however, that alternative GR ligands—dexamethasone, fludrocortisone, and triamcinolone have not been shown to elicit anti-platelet effects under the experimental conditions used in these studies.^27^^,^^28^ This difference in activation is thought to be due to the formation of a heterodimeric complex between GR and the mineralocorticoid receptor that is susceptible to the differential activation by specific receptor ligands. The mechanism underlying the negative regulation of secondary mediator signalling by GR and its ligand prednisolone is yet to be fully explored although evidence suggests that this might be mediated through the regulation of signalling events downstream of the P2Y_12_ receptor.^28^

### 3.2 Oestrogen receptor

Oestradiol-17β (E2) and ERs are not only well known for their role in reproductive and sexual development but also known to directly influence cardiovascular health.^53^ Human platelets have been shown to express ERβ but not ERα.^22^ Studies investigating the effects of several forms of oestrogen, including oestrone (E1), oestradiol (E2), and oestriol (E3) on platelet function have yielded conflicting results. In one study, acute treatment of platelets *ex vivo* with either E1 or E3 was found to increase aggregation to adrenaline or ADP.^24^ In contrast, chronic treatment with oestrogen, in an alternative study, investigating oestrogen replacement therapy (3 months), found a significant decrease in adrenaline-induced platelet aggregation and ATP release in patients receiving the therapy compared to the control groups.^25^ In further support of this, chronic treatment with high levels of oestradiol in mice was found to cause a marked decrease in platelet responsiveness both *ex vivo* and *in vivo*, with both an increase in bleeding time and resistance to thromboembolism being observed.^26^ However, it is important to note these effects on platelet reactivity are due to modulation of expression of platelet proteins (such as β1 tubulin) during haematopoiesis that then alter platelet production and activation,^26^ rather than a direct non-genomic effect on platelet function.

### 3.3 Androgen receptor

The AR, activated by either testosterone or dihydrotestosterone, has been identified in platelets,^22^ but little is known regarding its potential role in the regulation of platelet function. Some studies have specified that the aggregation response of platelets isolated from male rats was stronger in comparison to female rats owing to higher levels of androgenic steroids^46^ as platelet aggregation was found to be reduced following castration in male rats and the reversal of these effects following treatment with testosterone.^47^ Pilo *et al.* also reported that acute treatment of rat or human PRP with testosterone potentiates platelet aggregation induced by ADP, adrenaline, collagen, arachidonic acid, and calcium ionophore indicating its rapid non-genomic responses.^48^ Two independent studies also confirmed that testosterone causes a significant increase in TXA_2_ receptor density on the platelet surface, thereby, indirectly increasing platelet responsiveness.^54^^,^^55^ However, inhibition of platelet aggregation has also been observed following treatment with testosterone, although this was found to be attributed to endothelial NO synthesis and therefore not necessarily a direct effect of testosterone on the platelet AR and platelet activity.^23^

## 4. Type II NRs

### 4.1 Liver X receptor

LXR receptors are implicated in the regulation of fatty acid, cholesterol, and glucose homeostasis. Endogenous ligands for the LXR receptors include oxysterols such as 22(R)-hydroxycholesterol, 24(S)-hydroxycholesterol, 27-hydroxycholesterol, and several synthetic ligands including GW3965 and T0901317 have also been developed.^56^^,^^57^ Like some of the other NRs, ligands for LXR have been described to have anti-inflammatory and atheroprotective properties, and LXRβ has been shown to be expressed in platelets.^31^ Treatment of platelets with the synthetic agonist GW3965 results in inhibition of platelet activation, with attenuation of aggregation, calcium mobilization, secretion and integrin activation observed following stimulation by collagen, collagen-related peptide (CRP-XL; a GPVI collagen receptor-specific agonist), and thrombin. In analysis of thrombosis, GW3965-treated mice were also found to form smaller, less stable thrombi following laser injury of the cremaster arterioles. LXR has also been shown to interact with several components of the GPVI signalling pathway following treatment with GW3965, including Syk and PLCγ2, and treatment with LXR ligands is associated with decreased phosphorylation and signalling.^31^ In support of this, another study reported the ability of endogenous LXR ligand 22(R)-OH-cholesterol [but not its stereoisomer 22(S)-OH-cholesterol] to inhibit collagen-induced platelet aggregation and shape change.^58^

**Figure 1:**
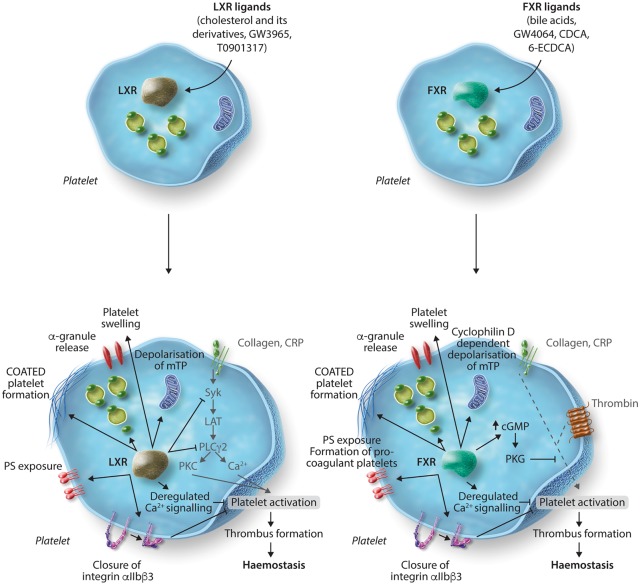
LXR and FXR ligands negatively regulate platelet function through inhibition of platelet signalling and formation of procoagulant coated platelets. Treatment of platelets with LXR ligands results in reduced tyrosine phosphorylation of key GPVI signalling molecules Syk, LAT and PLCγ_2_. An increase in the level of LXR–Syk and LXR–PLCγ_2_ interaction is also observed. Exposure to LXR ligands also renders platelets into a procoagulant state characterized by the exposure of phosphatidylserine and α-granule contents on the platelet surface, which is coupled with depolarization of the mitochondrial membrane potential, reduced calcium mobilization and down-regulation in the affinity of integrin α_IIb_β_3_, ultimately resulting in the inhibition of platelet aggregation. Similarly, incubation of platelets with FXR ligands can lead to platelet swelling and conversion to procoagulant coated platelets which is dependent on cyclophillin D activity. Additionally, FXR ligands are able to increase cGMP levels that promotes the activity of PKG and phosphorylation of VASP S239 and thereby suppresses platelet activation.

During thrombus formation, two distinct populations of platelets appear, coaggregated platelets, which support thrombus growth, and loosely attached procoagulant platelets that expose phosphatidylserine and support coagulation. Conversion to the procoagulant state is also thought to be associated with platelet hyper-reactivity, a trait often observed in patients with an increased risk of thrombosis including those with pathological conditions, such as hyperlipidaemia, obesity, and high plasma cholesterol levels. Treatment of platelets with LXR ligands, GW3965 and T0901317, and natural ligands, 27-OH-cholesterol and 24-(S)-hydroxyl-cholesterol, has also been shown to cause platelet inhibition to several agonists through the conversion of platelets to procoagulant coated platelets.^39^ LXR ligand-stimulated coated platelets not only expose phosphatidylserine at the membrane surface but also retain high levels of fibrinogen (which is converted to fibrin) and other alpha granule components at the platelet membrane (*Figure 1*). Conversion to the coated platelet state is thought to support coagulation but renders the platelet, through closure of integrin αIIbβ3, unresponsive to platelet agonists, which was also observed in platelets following treatment with LXR agonists. The mechanism by which this occurs in LXR agonist (GW3965)-treated platelets appears to be via deregulation of intracellular calcium signalling, depolarization of the mitochondrial membrane potential independently of cyclophillin D, and generation of reactive oxygen species (ROS).^39^ It is therefore possible that the platelet dysfunction observed in patients with high cholesterol, hyperlipidaemia, metabolic syndrome, and obesity could be attributed to altered LXR signalling in platelets.

### 4.2 Farnesoid X receptor

The bile acid receptor, FXR, which is recognized to regulate bile acid and cholesterol homeostasis has been identified in both human and mouse platelets. Treatment of platelets with synthetic FXR ligand GW4064 was found to cause a decrease in sample turbidity,^29^^,^^39^ which was later confirmed to be due to platelet swelling and conversion of platelets to a procoagulant state, forming coated-platelets.^39^ Synthetic and natural FXR ligand-dependent formation of coated platelets, prior to platelet agonist stimulation, results in phosphatidylserine exposure, retention of fibrinogen, fibrin and alpha granule proteins at the platelet surface, cyclophillin D-dependent depolarization of the mitochondrial membrane, sustained calcium signalling, generation of reactive oxygen species, and closure of integrins at the platelet surface.^39^ This closure of platelet integrins is believed to underlie the observed reduction in platelet aggregation to platelet agonists. Although the initial kinetics of thrombus formation was increased in mouse *in vivo* models of thrombosis, consistent with a procoagulant state, thrombus stability was significantly decreased following treatment with the FXR ligand GW4064, in agreement with reduced integrin function, which is essential for stable thrombus formation.^29^ Treatment with FXR ligands was also found to be associated with an increase in intracellular levels of cGMP in platelets, indicative of deregulation of intracellular signalling (*Figure 1*). Platelets from FXR-deficient mice were found to be unresponsive to the actions of FXR agonists, confirming the selective non-genomic actions of these ligands to the FXR.^29^

### 4.3 Peroxisome proliferator-activated receptors

PPARs represent three NR isoforms, PPARα, PPARβ, and PPARγ, which are involved in cell development, differentiation, cholesterol and fatty acid metabolism, and glucose homeostasis. All three isoforms of PPARs, upon binding to their ligands, are capable of heterodimerizing with the RXR^43^ and all have been identified to have acute, non-genomic, negative-regulatory effects in human platelets.

#### 4.3.1 PPARα

**Figure 2:**
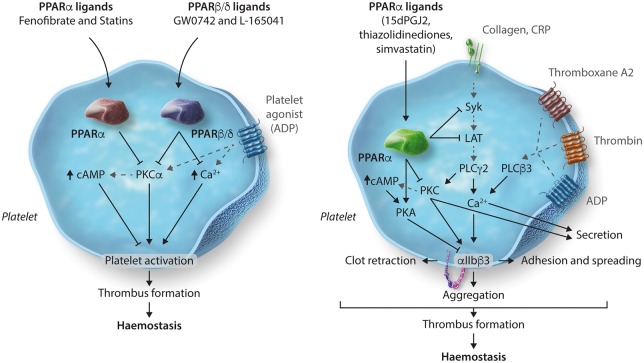
Non-genomic regulation of platelets by PPAR ligands. PPARα ligands, fenofibrate or simvastatin and PPARβ/δ ligands, GW0742 and L-165041 cause a reduction in intracellular calcium mobilization and platelet activation. This inhibition was found to be mediated by augmented levels of cAMP and attenuation of PKCα through its interaction with PPARα or PPARβ/δ, which limits its availability to facilitate signalling downstream of PKCα. Treatment with PPARγ ligands inhibits platelet activation to collagen through an inhibition in phosphorylation of Syk and LAT that mediate signalling initiated by the collagen receptor GPVI. Negative regulation of integrin α_IIb_β_3_ outside-in signalling was observed as an outcome of up-regulation of PKA activity and inhibition in phosphorylation of β3 and subsequent downstream signalling molecules—Syk, PLCγ_2_, PKC substrates, FAK and PI3K substrates.

The treatment of platelets with ligands of PPARα such as fenofibrate or statins (simvastatin) has been shown to inhibit ADP-stimulated platelet activation by increasing intracellular levels of cAMP via a PPARα-dependent mechanism. In support of this, the observed inhibition can be reversed following treatment with PPARα antagonist GW6471.^33^ This dependence on PPARα is further reinforced by experiments that show fenofibrate-induced inhibition of platelet activation and increased bleeding time in mice does not occur in mice deficient in PPARα. Fenofibrate-induced inhibition of platelet activity was found to be mediated through up-regulation of cAMP levels via inhibition of PKCα, a key mediator of platelet signalling, through interaction between PPARα and PKCα. This interaction is believed to sequester PKCα away from its substrates and thereby attenuates platelet functions (*Figure 2*).^33^ These findings identify PPARα as a key mediator of statin and fenofibrate-mediated anti-platelet activity.

#### 4.3.2 PPARβ/δ

Studies using synthetic ligands for PPARβ/δ, GW0742, and L-165041 have identified negative-regulation of platelet activity arbitrated through PPARβ/δ. Incubation with PPARβ/δ ligands showed inhibition of platelet aggregation and mobilization of intracellular calcium following stimulation by several platelet agonists.^34^ PPARβ/δ can also be activated by the prostaglandin PGI_2_, and therefore some of the inhibitory effects of PGI_2_ on platelet activity could also be mediated through PPARβ/δ in addition to the prostaglandin IP receptor but this has yet to be tested.^34^ Similar to PPARα, treatment of platelets with synthetic ligands of PPARβ/δ have been shown to cause an increase in intracellular cAMP levels and PKCα has been identified as a potential binding partner of the receptor indicating a plausible mechanism by which PPARβ/δ regulates platelet reactivity (*Figure 2*)^35^ PPARβ/δ ligands have been shown to decrease plaque formation and attenuate the progression of atherosclerosis.^59^ As platelets play a key role in the initiation and progression of atherosclerosis, antiplatelet effects of PPARβ/δ ligands may partly explain such observed reduction in the development of atherosclerosis.

#### 4.3.3 PPARγ

PPARγ is the most widely studied of the PPAR family in platelets. This is mainly because of its direct involvement with numerous cardiovascular diseases, such as diabetes mellitus, atherosclerosis, and thrombosis.^60–62^ Synthetic ligands of PPARγ, the thiazolidinediones (pioglitazone, rosiglitazone, lobeglitazone, etc.), are currently in use for the treatment of type 2 diabetes and have been observed clinically to have cardio-protective properties. The anti-platelet activity of PPARγ ligands may provide a mechanistic basis that in part underlies these observations. For example, a clinical study conducted on patients suffering from coronary heart disease and taking rosiglitazone reported its long-term antiplatelet effects with down-regulation of P-selectin exposure and granule secretion.^63^ Exposure of platelets *ex vivo* to the endogenous (15d-PGJ_2_) and synthetic (rosiglitazone and ciglitazone) ligands of PPARγ has been shown to inhibit platelet activation to a variety of platelet agonists, including the G-protein-coupled receptor agonists—thrombin and ADP,^32^ GPVI agonists—collagen and CRP-XL,^38^ and the adhesion receptor integrin α_IIb_β_3_ agonist fibrinogen.^39^ PPARγ ligands, 15d-PGJ_2_ or rosiglitazone, inhibit platelet responses including granule secretion and TxB_2_ synthesis in response to thrombin or ADP.^32^ These ligands have also been shown to reduce GPVI agonist-stimulated platelet aggregation, granule secretion, and mobilization of intracellular calcium, via inhibition of early GPVI signalling events such as phosphorylation of Syk and LAT.^38^ PPARγ was also found to interact with Syk and LAT upon stimulation with collagen in the absence of PPARγ ligands; however, this interaction is disrupted on treatment with PPARγ ligands. These ligands have also been shown to inhibit integrin α_IIb_β_3_ outside-in signalling through the up-regulation of PKA activity. PPARγ ligand-dependent inhibition of β3 phosphorylation and other downstream signalling molecules of the integrin α_IIb_β_3_ signalling pathway including Syk, PLCγ_2_, PKC, FAK, and PI3K indicates several different mechanisms by which PPARγ ligands can negatively regulate platelet function.^39^ This negative regulation of platelet activity has also been found to result in an inhibition of thrombus formation *in vivo* in animal models following treatment with another synthetic PPARγ ligand, pioglitazone.^37^

PPARγ is also implicated in a mechanism by which statins mediate acute anti-platelet effects.^33^^,^^36^ Treatment of human whole blood with simvastatin has been shown to cause a reduction in platelet aggregation to ADP. This inhibition of platelet function was attributed to an increase in intracellular cAMP levels which is associated with PPARγ activity and its association with and inhibition of PKCα (*Figure 2*).^33^ In addition, treatment of platelets with simvastatin was also found to inhibit collagen-induced platelet aggregation, granule secretion, integrin activation, and Ca^2+^ mobilization in a PPARγ-dependent manner. This was found to involve PPARγ-dependent mediation of mitogen-activated protein kinase (MAPKs, i.e. p38 MAPK, ERK) signalling by increasing association of MAPKs with the receptor resulting in an increase of cAMP formation that is associated with an increase in VASP Ser157 phosphorylation and inhibition of Akt phosphorylation.^36^

### 4.4 Retinoic acid receptor

**Figure 3:**
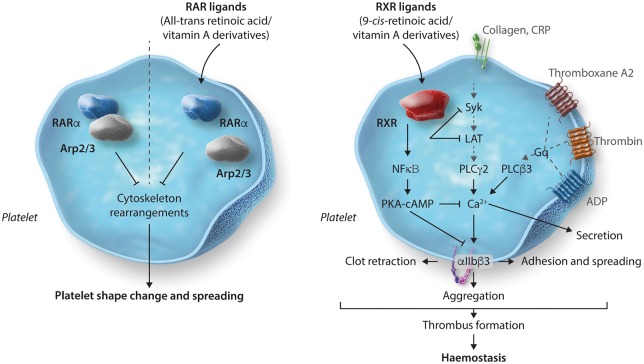
Inhibition of platelet function by RAR and RXR ligands is mediated through Arp2/3- and Gq-induced Rac activation and up-regulation of PKA activity, respectively. RAR ligand atRA disrupts the RARα–Arp2/3 interaction resulting in an inhibition of cytoskeletal rearrangements and platelet spreading. RXR ligands, 9cRA and methoprene acid inhibit platelet activation to a range of platelet agonists that include GPCR agonists (ADP, U46619 or thrombin) and GPVI agonists (collagen or CRP-XL). Interaction of RXR with Gq and subsequent negative regulation of Rac activation is one of the probable explanations for the reduction in GPCR-mediated platelet activation. These ligands have also been shown to up-regulate PKA activity in a cAMP- and NFκβ-dependent manner providing a more generalized mechanism of inhibition.

RARs play a critical role in numerous biological processes, including development, reproduction, immunity, organogenesis, and homeostasis.^64^ Three forms of RAR exist—RARα, RARβ, and RARγ. Of these, RARα is ubiquitously distributed and has been reported to be robustly expressed in human platelets, whilst the other two isoforms have tissue-specific distribution.^65^ RARs are activated by retinoids which are metabolites of vitamin A and several synthetic ligands also exist.^64^ In platelets, RARα has been observed to directly interact with actin-related protein-2/3 complex (Arp2/3) subunit 5 (Arp2/3s5) which is required for the regulation of platelet cytoskeletal processes. Treatment of platelets with the endogenous RARα ligand all-*trans*-retinoic acid (atRA) disrupts the RARα–Arp2/3 interactions resulting in an inhibition of both cytoskeletal rearrangements and platelet spreading (*Figure 3*).^41^ Recent developments have reported that RARα is capable of regulating protein synthesis (including microtubule-associated protein-1 light chain 3 beta 2) in human platelets by binding to a subset of mRNAs and blocking translation. Schwertz *et al.*^66^ found that platelets treated with RARα ligand atRA for several hours displayed significantly altered levels of protein synthesis compared to controls.

### 4.5 Retinoid X receptor

RXR is regarded as one of the most important receptors in this superfamily. Most likely due to its ability to interact with almost a quarter of the known human NRs (PPAR’s, LXR, FXR, PXR, etc.) and form heterodimers,^67^ although, the presence of RXR homodimers has also been reported.^68^ RXR is well characterized and is involved in the regulation of some of the most vital and fundamental biological processes including cell proliferation, differentiation and death, haematopoiesis, metabolism (glucose, fatty acid, and cholesterol), and pattern formation during embryogenesis.^69^ Human platelets have been shown to express RXRα and RXRβ (but the presence or absence of RXRγ has not been established) and are known to form heterodimers with PPARα, PPARγ, and LXR in platelets.^43^ Treatment of platelets with the endogenous ligand of RXR, 9-*cis*-retinoic acid or the synthetic ligand, methoprene acid, results in inhibition of platelet function stimulated by Gq-coupled GPCRs—ADP, U46619^42^ or thrombin and also, GPVI-mediated platelet activation via stimulation by collagen or CRP-XL.^43^ Regulation of GPCR-mediated platelet activation by RXR has been associated with its binding to Gαq in a ligand-dependent manner that inhibits Gq-induced Rac activation and intracellular Ca^2+^ mobilization.^42^ Exposure to RXR ligands has also been shown to reduce integrin α_IIb_β_3_ outside-in signalling and cytoskeletal rearrangements. The negative regulation of several platelet activation pathways and processes results in robust inhibition of thrombosis and haemostasis *in vivo.*^43^ As seen with several other NRs, treatment with RXR ligands has also been shown to up-regulate PKA activity and VASP S157 phosphorylation via a process that is dependent on cAMP and also involves NFκβ (*Figure 3*). This suggests that RXR ligands inhibit platelet function using several different inhibitory mechanisms which is likely to reflect its ability to form heterodimers with several different NRs in platelets.^43^

### 4.6 Vitamin D receptor

The VDR is another ligand-activated transcription factor that mediates the actions of vitamin D and its metabolites. VDR is also known to form a heterodimer with the RXR and regulate calcium homeostasis, cell growth and differentiation, detoxification of xenobiotics, and modulation of adaptive and innate immunity.^70^ Although anticoagulant effects of vitamin D have been reported and VDR signalling has been characterized in monocytes and vascular cells, the role for the VDR in platelet function remains unknown. Human platelets have been found to express the VDR. Biochemical fractionation studies along with immuno-electron microscopy analysis identified the VDR to be localized in the soluble and mitochondrial compartment.^44^ Although little is known about the role for vitamin D and the VDR in platelet function, a patient study identified a strong association between low vitamin D plasma levels and a high mean platelet volume, a marker of platelet hyperactivity.^45^

## 5. Future perspectives

### 5.1 Could NRs offer anti-platelet therapeutic targets?

The role of platelets in controlling haemostasis and initiating thrombosis is well known. This makes them important therapeutic targets for the treatment of cardiovascular diseases, particularly atherothrombosis.^71^ Despite significant advances in the development of antithrombotic therapeutics, they are associated with increased bleeding risk and their efficacy is often compromised in patients suffering from several conditions, such as hypertension and diabetes.^11^^,^^72^^,^^73^ Therefore, more refined and effective therapeutics that ensure a balance between the treatment of thrombosis and related complications is needed.

A key step forward would be using our current knowledge of the molecular mechanisms governing platelet functions as the basis for the development of more effective and safer anti-platelet therapies. Both natural and synthetic ligands of NRs have been shown to exhibit non-genomic effects to alter platelet function through a variety of mechanisms, several of which appear to be shared by different NR family members.^21^^,^^74^ For instance, RXR, FXR, PPARα, PPARβ, and PPARγ receptors have been shown to be involved in the regulation of platelet inhibitory signalling pathways by either increasing cAMP or cGMP levels or directly modulating PKA/PKG activity. LXR and PPARγ can negatively regulate signalling downstream of the collagen receptor via interactions with different components of the GPVI signalling cascade. Given the potential of heterodimeric receptor interactions between RXR and other NRs, the idea of cross-talk becomes even more pronounced.^43^

### 5.2 Important considerations

Development of NRs as anti-platelet therapeutic targets requires a few important considerations. First, the majority of the studies conducted so far focus on understanding the acute effects of NR ligands on platelet function; therefore, prior to further development it would be important to study the implications of chronic exposure of platelets to NR ligands. As NRs can regulate the expression of multiple genes, in various cell types it is therefore highly likely that chronic exposure to NR ligands could lead to systemic effects that might indirectly affect platelet function.^75^

Secondly, although, there exists a clear distinction between genomic and non-genomic effects, the existence of mRNA in platelets and their limited ability to perform translation raises the possibility^17^^,^^76^ that there are interactions between NRs and mRNA in platelets as in nucleated cells. While the differences in the timescales taken to elicit these genomic-like effect (hours) in comparison to the non-genomic effects (minutes) still enable the differentiation between the two regulatory mechanisms. Future studies should consider including inhibitors of translation which will help further differentiate between genomic and truly non-genomic actions of these receptors, or indeed determine whether NR ligands are capable of regulating protein translation in platelets and characterizing whether any changes in protein levels have functional effects. Schwertz *et al.*^66^ recently described such a mechanism demonstrating RARα-dependent translational control in human platelets, which resulted in the synthesis of several transcripts. Whether other NRs (such as RXR and PPARs), identified in platelets, can also replicate such a mechanism is still unknown. Moreover, evaluating whether genomic and non-genomic regulation can facilitate cross-talk between the different NRs in platelets requires further investigation. Development of NRs as anti-platelet therapies would require careful balancing of their genomic vs. non-genomic effects not only in platelets but also systemically.

Finally, it is important to note that NRs share a significant level of structural similarity with each other, making them potentially promiscuous in nature.^77–81^ Studies examining the genomic roles for the NRs have shown, for example that 15d-PGJ_2_ is an endogenous ligand for PPARγ but it can also act as an antagonist for FXR^82^ and phytanic acid has the ability to activate both PPARα and RXR.^83^ Guggulsterone is regarded as an ER agonist but an antagonist to FXR, GR, and AR,^84^ although it does not appear to function as a non-genomic FXR antagonist in platelets (LA.M, A.J.U, J.M.G, unpublished observations). Similarly, LG100754 is a highly specific RXR: PPARγ agonist while it acts as a strong antagonist of RXR homodimers.^85^ This makes the selective targeting of the NRs even more challenging and as such identification of ligands that function in a receptor- and gene-specific manner is important. Future work will be required to establish how and when NR heterodimers regulate platelet activity, and for each NR to establish its role in normal physiological processes.

## 6. Conclusions

Platelets are known to act as direct contributors towards the progression of CVDs such as atherosclerosis,^86^ and their activity becomes considerably enhanced in cases of hyperlipidaemia,^87^ obesity,^88^ diabetes mellitus,^89^ or hypertension.^90^ Many NRs have been found to be expressed in human platelets, including AR, ER, GR, FXR, LXR, PPARs, RAR, RXR, and VDR, and agonists for several of these receptors have been shown to elicit anti-platelet effects by a variety of mechanisms. NRs including PPARs, LXR, and FXR ligands have all been reported to have anti-atherosclerotic effects,^91^^,^^92^ coupling this with their anti-platelet effects; there exists the possibility of a potentially new paradigm of treatment that can target a range of pathophysiological conditions whilst also offering platelet-targeted anti-thrombotic activity. Of FDA-approved drugs, 13% function by targeting NRs for the treatment of numerous pathological conditions^93^ (*Table 3*), and as a result, the effects on platelets might be a likely consequence associated with the administration of these drugs. It is important for the future development and use of NR agonists that their acute and long-term effects on platelet function are fully understood.

**Table 3:** Commercially available nuclear receptor drugs

Nuclear receptor	Disease	Drug generic name (marketed drug)
GR	Metabolic and immunological Disorders	Dexamethasone (Dexasone), Prednisolone (Orapred)^94^^,^^95^
ER	Breast cancer, obesity	Tamoxifen (Nolvadex), Raloxifene (Evista)^96^^,^^97^
PPARα	Dyslipidaemia, atherosclerosis	Fenofibrate (Tricor)^98^
PPARγ	Diabetes, obesity	Pioglitazone (Actos), Rosiglitazone (Avandia)^99^^,^^100^
RAR	Leukaemia, acne	13-*cis*-retinoic acid (Isotretinoin)^101^
RXR	Leukaemia, Kaposi sarcoma, eczema	9-*cis*-retinoic acid (Alitretinoin), Bexarotene (Targretin)^102–104^
VDR	Osteoporosis, calcium homeostasis	Calcitriol (Calcijex), Paricalcitol (Zemplar)^105^^,^^106^

## Authors’ contributions

A.J.U, G.D.F, and J.M.G wrote the review. A.J.U and G.D.F contributed equally.

**Conflict of interest:** none declared.

## Funding

This work was supported by the British Heart Foundation (RG/15/2/31224) and a Felix Scholarship.
